# Quantifying the effects of vagus nerve stimulation on gastric myoelectric activity in ferrets using an interpretable machine learning approach

**DOI:** 10.1371/journal.pone.0295297

**Published:** 2023-12-01

**Authors:** Mahmoud Zeydabadinezhad, Charles C. Horn, Babak Mahmoudi

**Affiliations:** 1 Department of Biomedical Informatics, Emory University, Atlanta, GA, United States of America; 2 Department of Medicine, University of Pittsburgh, Pittsburgh, PA, United States of America; 3 Department of Biomedical Engineering, Georgia Institute of Technology and Emory University School of Medicine, Atlanta, GA, United States of America; Feinstein Institute for Medical Research Fertility Research Laboratory: Northwell Health Feinstein Institutes for Medical Research, UNITED STATES

## Abstract

Vagus nerve stimulation (VNS) is a potential treatment option for gastrointestinal (GI) diseases. The present study aimed to understand the physiological effects of VNS on gastrointestinal (GI) function, which is crucial for developing more effective adaptive closed-loop VNS therapies for GI diseases. Electrogastrography (EGG), which measures gastric electrical activities (GEAs) as a proxy to quantify GI functions, was employed in our investigation. We introduced a recording schema that allowed us to simultaneously induce electrical VNS and record EGG. While this setup created a unique model for studying the effects of VNS on the GI function and provided an excellent testbed for designing advanced neuromodulation therapies, the resulting data was noisy, heterogeneous, and required specialized analysis tools. The current study aimed at formulating a systematic and interpretable approach to quantify the physiological effects of electrical VNS on GEAs in ferrets by using signal processing and machine learning techniques. Our analysis pipeline included pre-processing steps, feature extraction from both time and frequency domains, a voting algorithm for selecting features, and model training and validation. Our results indicated that the electrophysiological changes induced by VNS were optimally characterized by a distinct set of features for each classification scenario. Additionally, our findings demonstrated that the process of feature selection enhanced classification performance and facilitated representation learning.

## Introduction

Electrical vagus nerve stimulation (VNS) is emerging as a potential therapy for gastric motility disorders [1]. However, the VNS mechanisms of action on gastric motility regulation has yet to be fully understood. This understanding is crucial for developing more effective therapies. Gastric electric activity (GEA) is known to be a physiological signal that regulates gastric motility and can be recorded by means of electrogastrography (EGG) [2,3]. EGG can be used as a feedback signal for closed-loop adaptive VNS interventions, however its adoption in clinical practice remains limited. The primary reasons for this underutilization include the intrinsic properties of GEA and the absence of standardized protocols for electrode placement, both of which constrain the clinical applicability when recorded non-invasively [4]. However, it is worth noting that recent studies [5,6] have demonstrated the successful fabrication of high spatial resolution EGG and Magnetogastrogram systems [7]. These advancements hold the potential to establish a clinical standard for EGG recording, ultimately facilitating its widespread adoption in clinical settings.

Pre-clinical and human studies have demonstrated that implanted electrodes can record GI myoelectric activities that contain significantly more information than skin surface electrodes [8]. Since the abdominal wall may have a low-pass filtering effect, the higher frequency information are attenuated in non-invasive EGG recordings [9]. These attenuated signal components are known to be associated with gastric contractions [10]. While invasive recordings provide physiological signals that contain more information, compared to that of non-invasive recordings, they pose several challenges. In studies with electrodes implanted in the GI system, the subjects are required to remain sedentary or under anesthesia during data recording [11]. Most EGG-based studies have been done in a controlled environment with the subject either instructed not to move or being anesthetized [12,13]. Although collecting data in a controlled and sedentary manner is useful for some studies, it cannot synthesize realistic situations, such as recording data in ambulatory and non-clinical settings. One potential application of ambulatory EGG recordings is studying motion sickness [14–16] and the effectiveness of administered therapies to prevent emesis [17].

In this study, we formulated a machine learning approach to quantify the effects of VNS on GEA using EGG signals that were recorded invasively from the surface of the stomach. While previous research [18,19] has demonstrated the impact of VNS on alterations in gastrointestinal activity, these investigations have not explored EGG features beyond dominant frequency [2] (DF) and its derivatives. In our present study, we have incorporated an expanded array of diverse features, drawing from those commonly employed in related fields such as electroencephalography (EEG) and electromyography (EMG), to represent a broad range of physiological properties beyond the standard DF. Using this approach, we aim to address two main questions: 1- Can we identify the electrophysiological effects of electrical VNS on EGG signals recorded in an invasive and non-sedentary manner? 2- Do the electrophysiological effects depend on the electrical VNS parameters?

## Results

**Fig 1:**
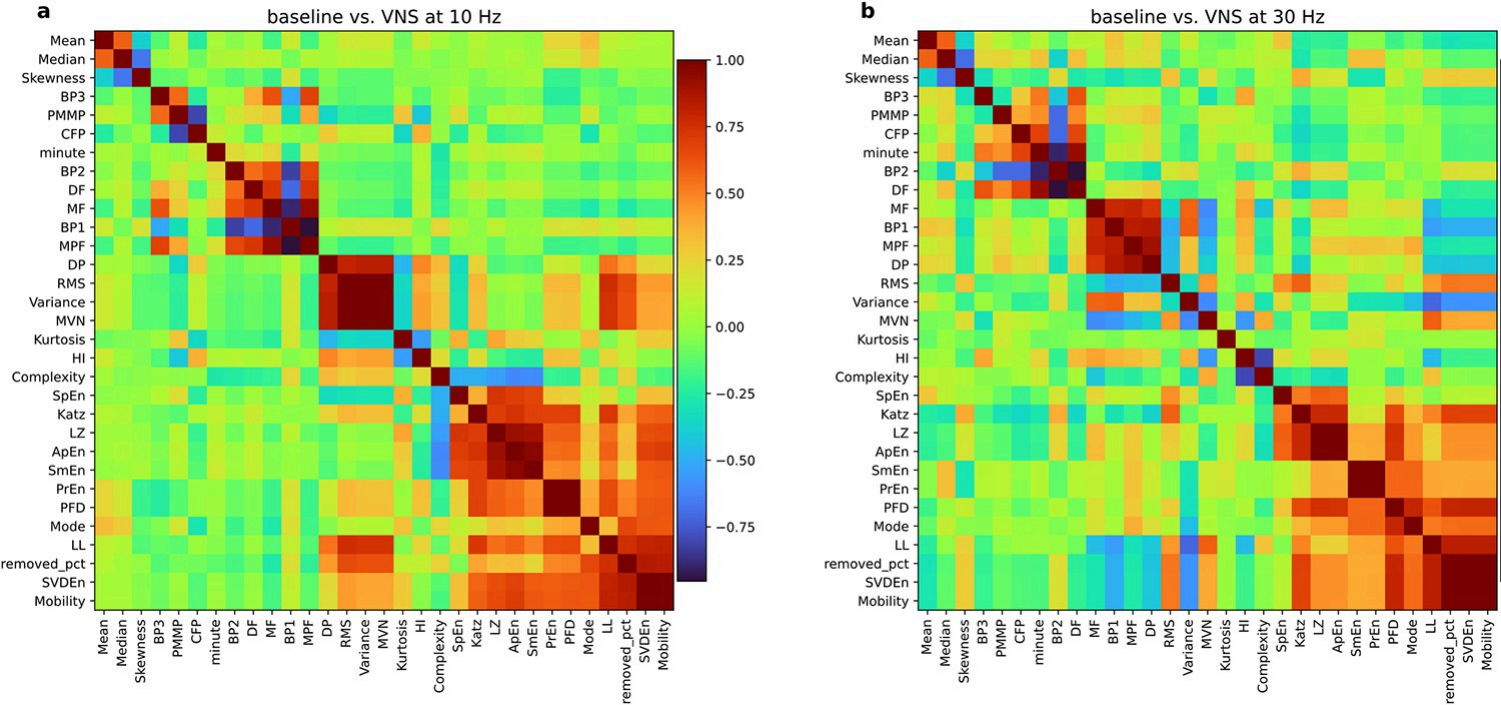
Correlation heatmap of the engineered features (See Table 1) **a**) baseline vs. VNS at 10 Hz **b**) baseline vs. VNS at 30 Hz. Each cell’s color shows to what extent features are correlated.

We considered two scenarios, i.e., baseline vs. VNS at 10 Hz and baseline vs. VNS at 30 Hz. One motivation for feature selection is first to find features that are correlated with each other and second, to remove those with high correlation from the analysis. Fig 1 presents the clustered correlation heatmaps of all features for two distinct scenarios: baseline versus VNS at 10 Hz and VNS at 30 Hz. This visual representation facilitates a comprehensive understanding of the relationships among features. In addition to the engineered features, we included ‘removed-pct’ which is the percentage of removed signal after applying the pre-processing steps. We only used samples with ‘removed-pct’, less than 30% for model training. From these heatmaps it is evident that there are clusters of features that are positively or negatively correlated with each other. The correlation heatmap in Fig 1A suggests a positive correlation between ‘removed-pct’ and features such as variance and RMS. This can be an indication of higher error in signal measurements due to physiologically implausible high signal values or abrupt changes in recorded signals, reflected in RMS and variance, respectively.

**Fig 2:**
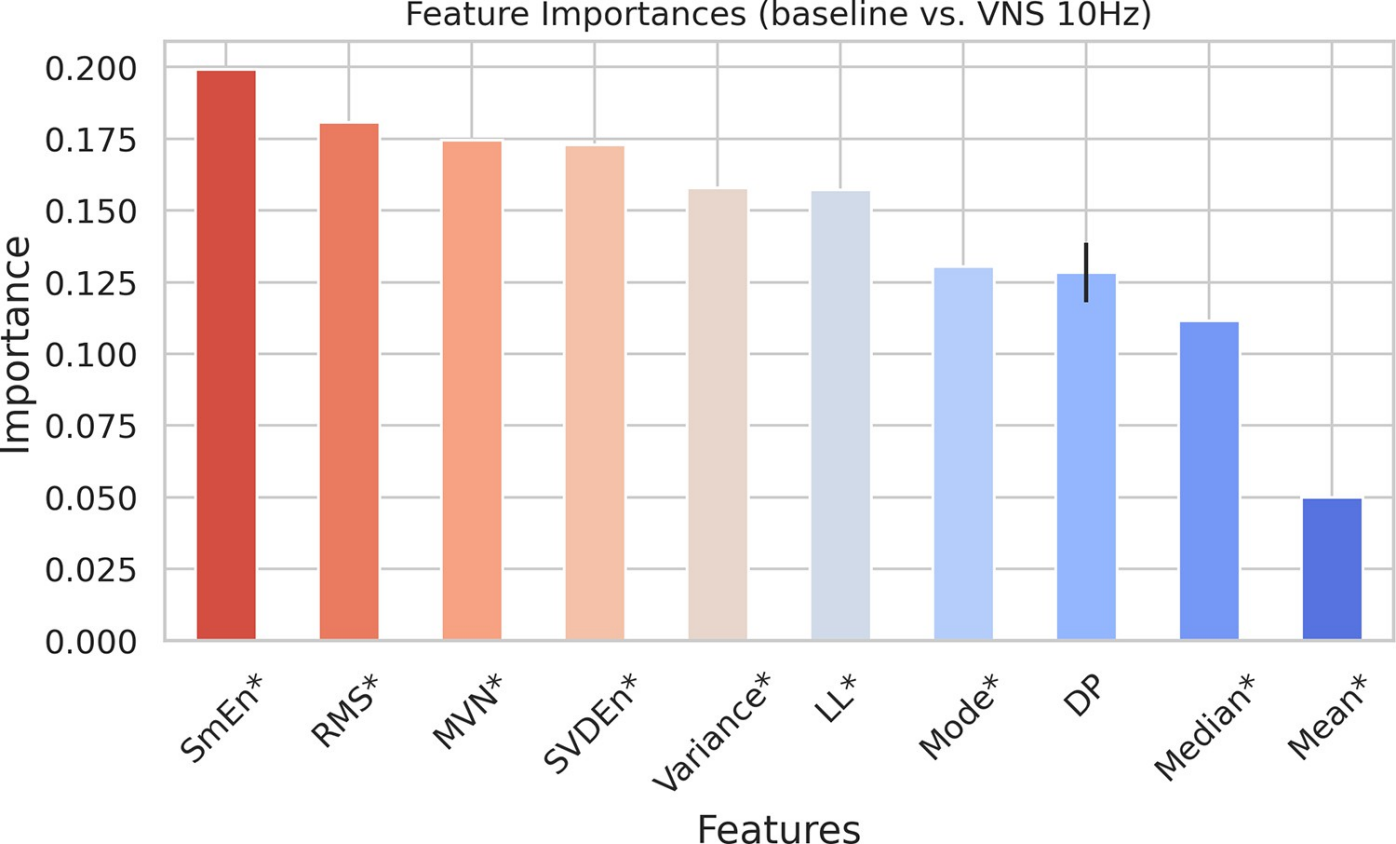
Selected features via our feature selection algorithm for baseline vs. VNS at 10 Hz (first scenario) and organized in a descending sequence of significance, as established by the Random Forest classifier. Error bars represent one standard deviation. ’*’ is used to show the time domain features. SmEn: Sample Entropy, RMS: Root Mean Square, MVN: (Entropy of) Multi-variate Normal, SVDEn: Singular Value Decomposition Entropy, LL: Line Length, DP: Dominant Power.

Fig 2 exhibits the features chosen for the first scenario, as identified by our feature selection algorithm, and organized in a descending sequence of significance, as established by the Random Forest classifier (See Methods). The Sample Entropy [20] (SmEn) of the signal emerged as the most significant feature, while Root Mean Square (RMS) was identified as the second most important attribute. RMS is a measure of the signal’s overall energy or amplitude [21], while Sample Entropy is a measure of the complexity or irregularity of a time series signal [22]. In the context of the first scenario, the differing Sample Entropy values between the baseline and VNS at 10 Hz suggest that the underlying dynamics or patterns of the EGG signals change because of VNS. Also, the variation in RMS could be indicative of the effect of VNS with 10 Hz frequency on the overall energy of EGG signals being analyzed. In general, most of the features selected in this scenario pertain to the signal’s amplitude (See S1 Fig).

**Fig 3:**
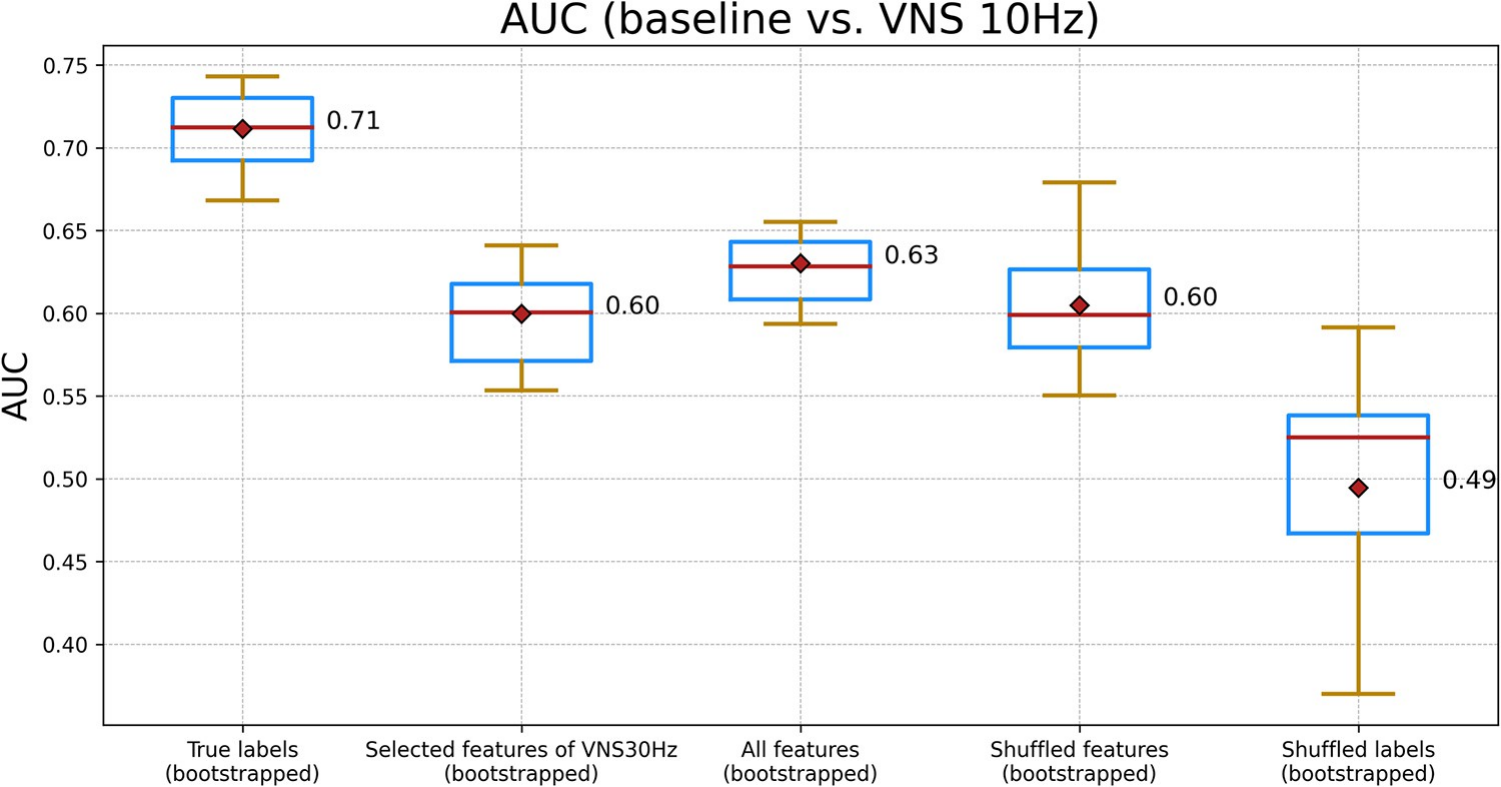
This illustration compares the AUC for a Random Forest classifier trained using various feature sets. From left to right: Features from Fig 2, features selected for VNS at 30 Hz, all features, a random selection of features equivalent in number to those in Fig 2, and randomly shuffled labels. The comparison is conducted in the context of the baseline versus VNS at 10 Hz scenario.

Utilizing the chosen features, as illustrated in Fig 2, a Random Forest classifier [23] was trained. Fig 3 demonstrates the Receiver Operating Characteristic—Area Under the Curve (ROC-AUC or AUC [24]) of this classifier, encompassing three distinct cases: training the classifier using the selected features, employing randomly chosen features for training, and utilizing randomly shuffled labels for the training process. We conducted a two-sample Kolmogorov-Smirnov (KS) test [25] to assess the null hypothesis that the AUC values, derived from a 5-fold cross validation (CV) of the classifier trained with the selected features from Fig 2, originate from the same distribution as the AUC values for the other two cases presented in Fig 3. The obtained p-values were < 0.001 (with test statistic of 1.0 and 0.872, respectively), enabling us to accept the alternative hypothesis that the AUC values for each case stem from distinct distributions (See S2 Fig for metrics other than AUC). These findings indicate that our feature selection algorithm played an important role in augmenting the performance of the classifier, thereby demonstrating its effectiveness. (See Methods for details)

**Fig 4:**
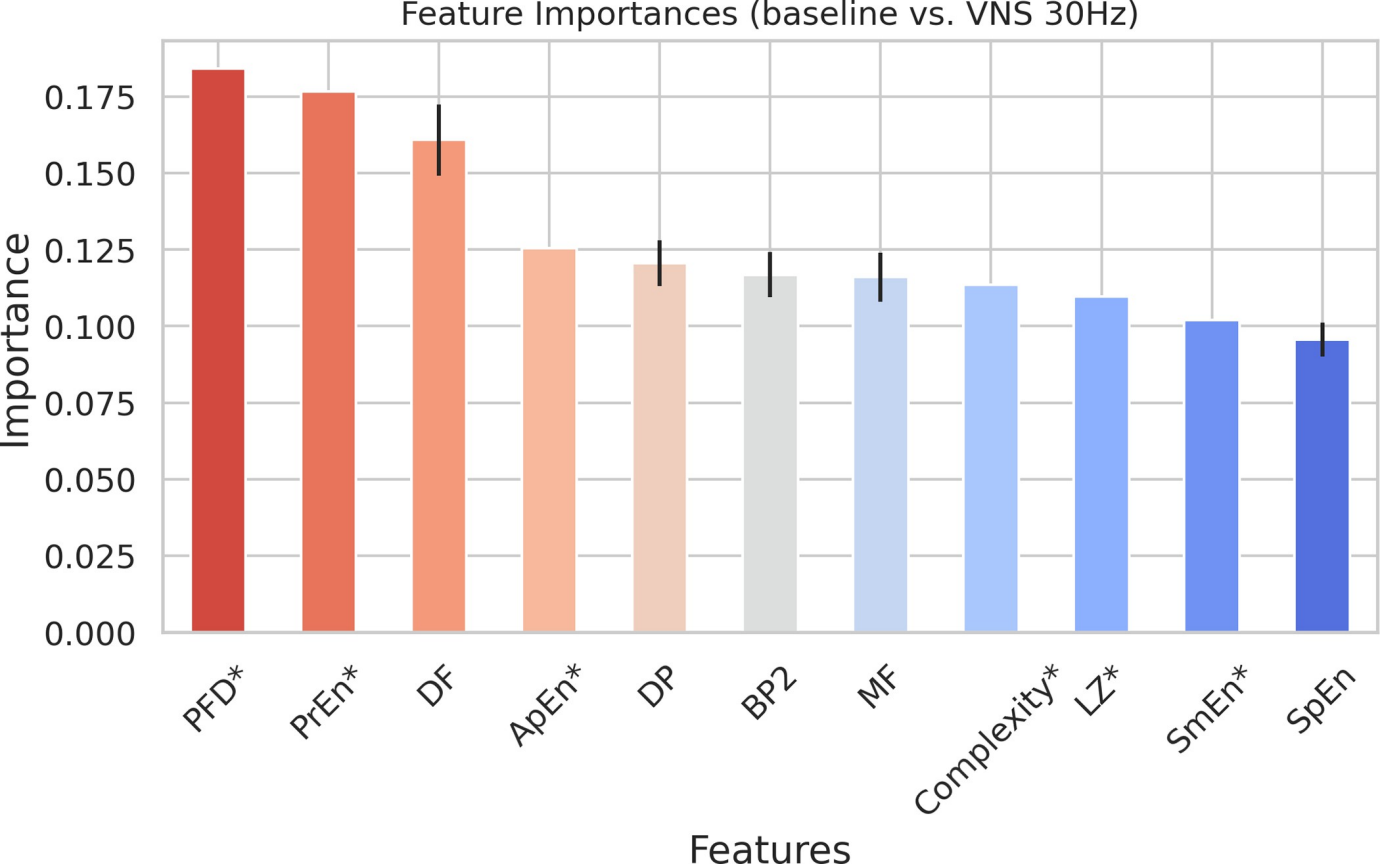
Selected features via our feature selection algorithm for baseline vs. VNS at 30 Hz (second scenario) and organized in a descending sequence of significance, as established by the Random Forest classifier. Error bars represent one standard deviation. ’*’ is used to show the time domain features. PFD: Petrosian Fractal Dimension, PrEn: Permutation Entropy, DF: Dominant Frequency, ApEn: Approximate Entropy, DP: Dominant Power, BP2: Band Power in 8–11 cpm, MF: Median Frequency, LZ: Lempel-Ziv Complexity, SmEn: Sample Entropy, SpEn: Spectral Entropy.

Contrary to the first scenario, Fig 4 reveals that the majority of the selected features for the second scenario, baseline vs. VNS at 30 Hz, are entropy and frequency based. This observation suggests that, in the case of VNS at 30 Hz, the changes induced by the VNS are more prominently reflected in the signal’s pattern or frequency content rather than its amplitude or energy (See S1 and S3 Figs). This distinction highlights the potential differences in the underlying mechanisms and effects of VNS at various frequencies, which may provide valuable insights into the physiological responses to stimulation. It is worth mentioning that the most important feature in the second scenario, Petrosian Fractal Dimension [26] (PFD), was originally introduced for the quantitative interpretation of epileptic EEG recordings [26,27] (See Appendix). Additionally, the presence of dominant frequency (DF), Dominant Power (DP), and normogastric band power (BP2) (See Table 1) in Fig 4, may align with the insights provided by entropy-based features (which measure the unpredictability or complexity of a signal), further showing that VNS at 30 Hz may demonstrate a greater impact on signal pattern and frequency shifts, rather than on signal amplitude or energy.

**Table 1:** List of time- and frequency-domain features used for EGG analysis. PMMP: Percentage of PSD that has higher value than DP/4. BP1-3: Relative band power between 3-8cpm, 8–11 cpm, and 11–15 cpm respectively.

Time domain	Frequency domain
Mean value	Dominant frequency (DF)[28]
Variance	Dominant Power (DP)[29]
Mode	PMMP [29]
Median	Spectral Entropy (SpEn)[30]
Skewness	BP1 [28]
Kurtosis	BP2 [28]
RMS	BP3 [28]
Line Length (LL) [31,32]	Crest factor of PSD [15,29]
Approximate Entropy (ApEn)[20,22]	Median frequency (MF)
Sample Entropy (SmEn) [20,22]	Mean power frequency [15,29]
Permutation Entropy (PrEn) [33]	
SVD Entropy (SVDEn) [34,35]	
Lempel-Ziv Complexity (LZ) [36]	
Hjorth Mobility & Complexity [37]	
Petrosian Fractal Dimension (PFD) [26,27]	
Hurst Index [38]	

**Fig 5:**
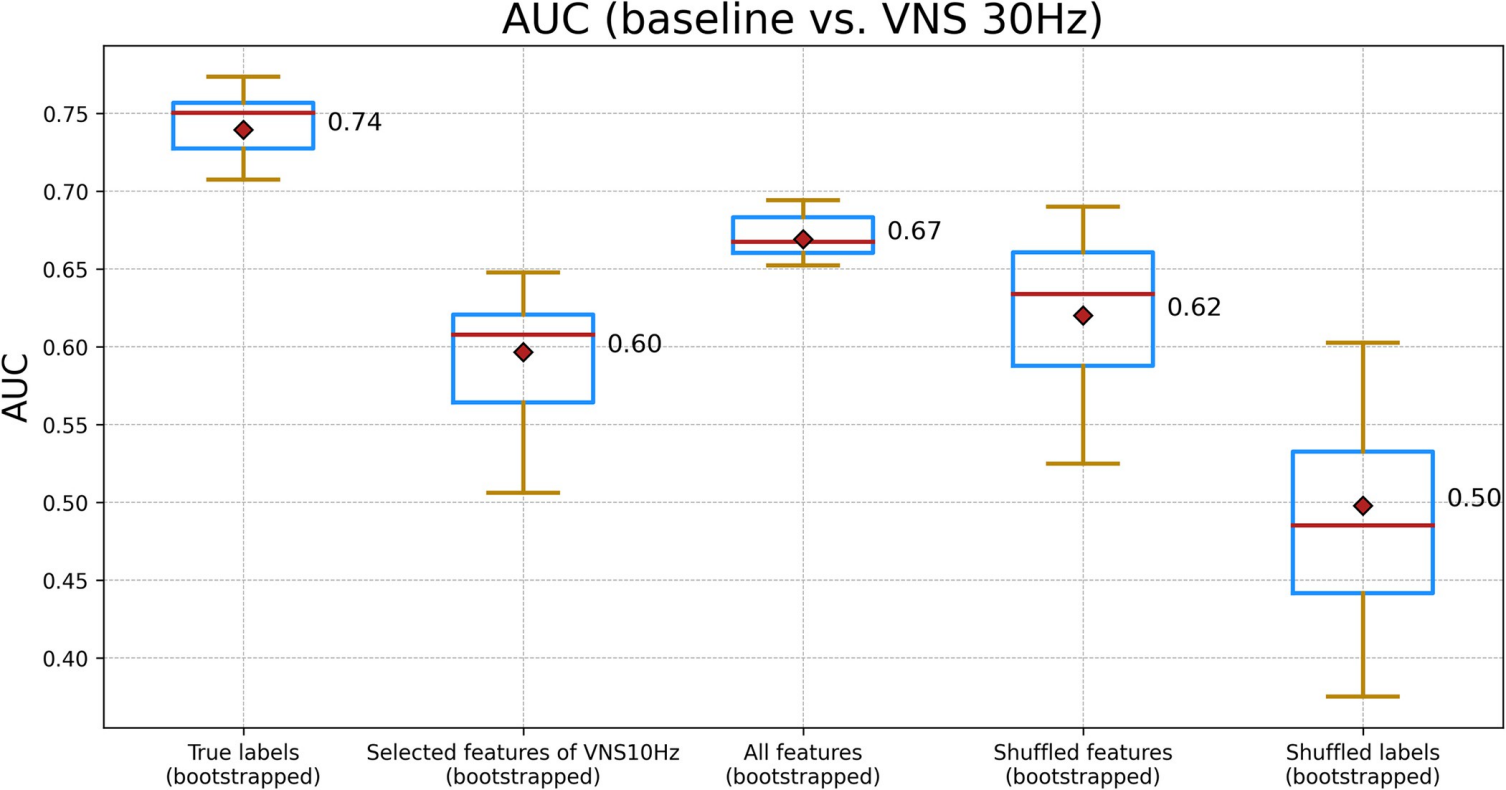
This illustration compares the AUC for a Random Forest classifier trained using various feature sets. From left to right: Features from Fig 4, features selected for VNS at 10 Hz, all features, a random selection of features equivalent in number to those in Fig 4, and randomly shuffled labels. The comparison is conducted in the context of the baseline versus VNS at 30 Hz scenario.

Fig 5 demonstrates the AUC values for a Random Forest classifier trained for the second scenario (See S4 Fig for metrics other than AUC). Similar to the first scenario, the AUC values derived from a 5-fold CV of the classifier trained with the selected features in Fig 4 were statistically significantly different from the other two cases showed in Fig 5 (two-sample KS test, test statistic: 0.948 and p-value< 0.001)

Taken together, the findings from Figs 2 to 5 indicate that, within the framework of our study, it is feasible to distinguish the effects of VNS on the EGG signals. Furthermore, the frequency of VNS may modulate the alterations observed in EGG, manifesting either as changes in signal amplitude and energy, or as shifts in signal complexity and frequency contents.

## Discussion

The main goal of this paper was to introduce a novel dataset and analysis pipeline to determine the effect of electrically induced VNS on EGG signals. Characterizing this effect is essential for better understanding the underlying physiological mechanisms of VNS for regulating the GI function and inform designing closed- loop GI-VNS systems. Our primary contribution in this study is to establish a robust methodological foundation for future research in this domain. While our dataset has its limitations, the methods and approaches we’ve introduced pave the way for more extensive and in-depth studies in the future.

Our study utilized a data acquisition schema that is rarely used due to the difficulties associated with surgery, electrode implantation, and long-term data collection. The advantages of this schema compared to cutaneous EGG were recording data that are not filtered by the abdominal wall and synthesizing a more realistic ambulatory setting. Disadvantages of the schema were loose electrode connections or abrupt movements of the ferrets causing artifacts in the recorded data. These artifacts were removed effectively by utilizing band-pass filtering in the pre-processing steps (See S5–S7 Figs). However, after removing the corrupted data portions, we were left with a small sample size, whereby 19.2% of the total collected data was discarded. Although dominant frequency (DF) is known to be the most widely used feature in EGG-related studies (See Methods), our preliminary analysis showed that DF alone was not informative enough to classify between baseline and VNS states in our data. To address this problem, we leveraged a machine learning approach. We employed a broader range of engineered features beyond DF to extract more information from our data. In exchange for these added features, we had to resolve another problem: the ratio between the number of samples (n) and the number of predictors or features (p). In our study, this ratio was close to 3. This can become problematic as most machine learning algorithms assume that there are many more samples than predictors [39] or p << n. In our case, the condition was exacerbated because the data was noisy, heterogeneous and could result in overfitting. There are multiple approaches [40] such as filter, wrapper, and embedded methods to handle datasets with too many features and a small number of observations. However, it is important to note that no single method is suitable for all datasets and situations [41]. To harness the power of each feature selection method, we devised a voting algorithm to rank the features selected by each single feature selection method and made a final decision based on majority voting. Our experiments demonstrated the efficacy of the voting algorithm, as evidenced by the enhancement in the AUC value of the trained classifier.

Moreover, the interpretability of the model was improved by reducing the total number of features utilized for classification and organizing them according to their importance. A model is considered interpretable if a human can comprehend the rationale behind its predictions [42]. Reducing the number of features and organizing them according to their importance contributes to interpretability, as it makes it easier to understand how each feature influences the prediction. Our analysis pipeline helped to demonstrate that we could programmatically distinguish between EGG signals recorded during baseline and VNS indicating that the electrophysiological effect of VNS on EGG signal can be identifiable. This finding is in accordance with previous research that investigated the effect of VNS on GI function and whether it changes the EGG signal [43]. To examine the influence of VNS frequency on the alterations observed in EGG, we employed the selected features for VNS at 10 Hz and 30 Hz. The selected features revealed that the impact of VNS at 10 Hz was predominantly noticeable in time domain features associated with signal amplitude and energy. Conversely, for VNS at 30 Hz, features pertaining to frequency content and entropy of signal were of greater significance. The observed differences in feature importance between the two scenarios highlight the potential variability in the underlying mechanisms and effects of VNS at different frequencies. While similar entropy features might be expected to have comparable importance, the distinct physiological responses to VNS at 10 Hz and 30 Hz can lead to variations in their relevance for classification.

There are limitations to our study in terms of data and methodology. The number of discarded samples was 33 and 44 for VNS at 10 Hz and 30 Hz, respectively. This 33% increment in discarded values in VNS at 30 Hz could be due to more loose electrode connection or a change in ferret movement patterns. Nevertheless, this difference in the quality of recorded signals during VNS at 10Hz and VNS at 30Hz could be a confounding factor and should be accounted for in future work. Another confounding factor is the state-dependency nature of EGG. For instance, two baseline recordings made of the same animal may differ if recorded in different sessions, based on how long before the recording session a ferret has ingested food. The same holds true for signals recorded during applying electrical VNS. A limitation associated with our methodology was that frequency domain features employed in this study are based on Fast Fourier Transform (FFT), however one assumption in FFT is that the input signal is periodic, but EGG is a non-stationary signal with chaotic properties. The future directions of our research will be focused on addressing the limitations of our data acquisition and analysis. The quality of collected data will be improved by more robust implantation of electrodes and employing wireless recording equipment. This will lead to a reduction in the number of invalid samples and more consistency in recording from different sessions. Regarding data analysis, we will adopt spectral analysis tools better suited for non-stationary signals such as wavelets and empirical mode decomposition [44] (EMD). These methods may provide a more accurate representation of EGG spectral information. Modern machine learning methods designed to generalize to Out-of-Distribution (OOD) data would offer an avenue to explore the state-dependent character of the recorded EGG and its inter- and intra-variability.

## Methods

### Data collection

**Fig 6:**
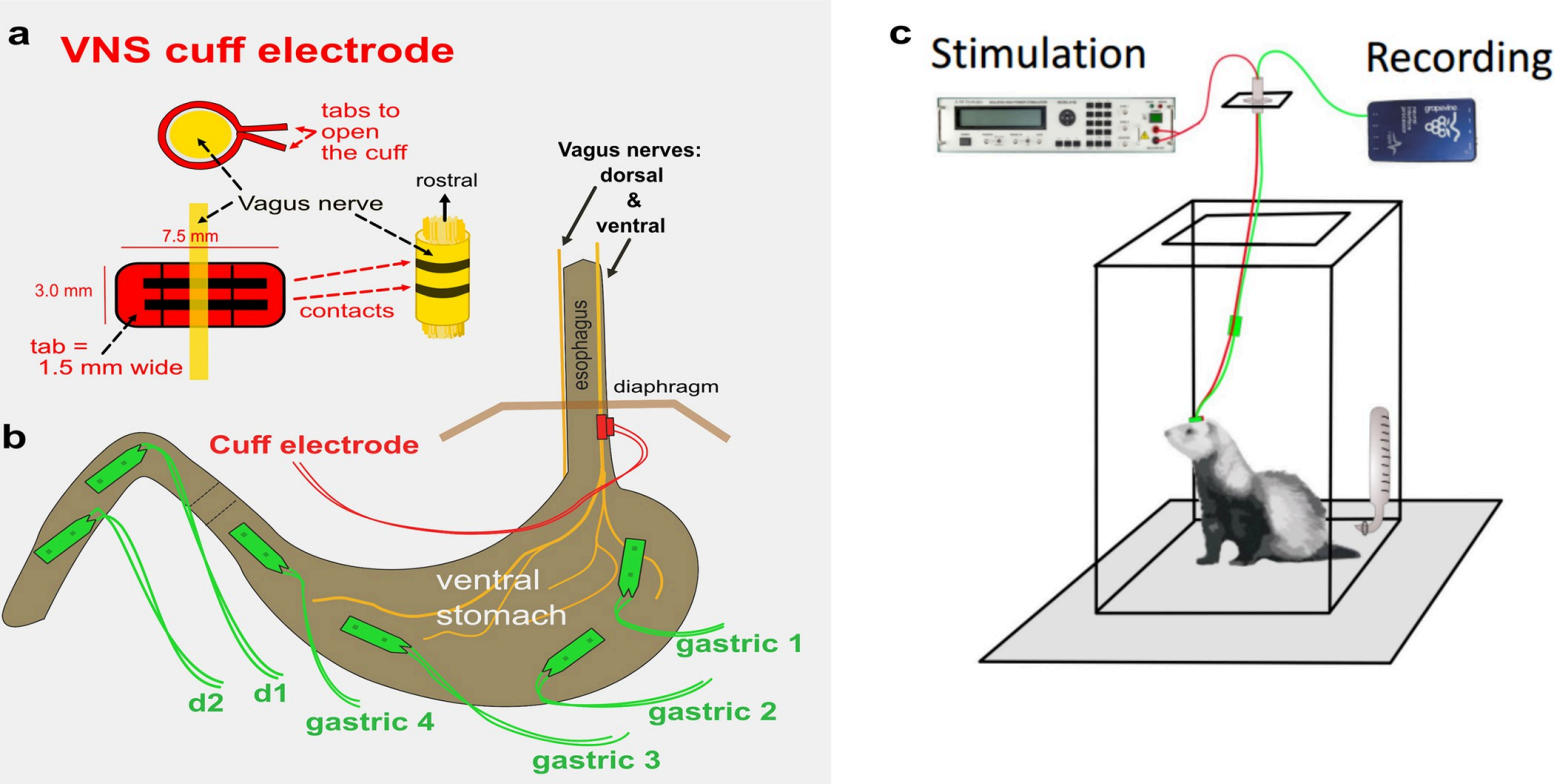
Electrode placements. **a**) show the structure of nerve cuff, **b**) position of surface electrodes (only ‘gastric 2’ was used in this study) and nerve cuff. **c**) A ferret’s head connection to the vagus nerve stimulator and EGG recording device.

Our data collection approach was designed to synthesize realistic ambulatory settings. We opted for a rarely practiced approach that involved implanting the VNS cuffs and EGG electrodes around the vagus nerve and on the serosal layer of the ferret stomachs, respectively. To study the physiological effects of VNS on the gastric function, our novel dataset was collected by recording EGG from the serosal layer of ferret stomach in two different conditions, i.e., before applying the VNS (baseline) and during application of the VNS. The recording was done in-vivo while the animals were awake and freely moving in their cage. To our knowledge, this is the first time that EGG signals were recorded in this manner. Under isoflurane anesthesia (1 to 3%), seven adult male ferrets were chronically implanted with vagus nerve cuffs and GI serosal surface electrodes. Surface electrodes were placed at four locations along the stomach axis (named gastric1 to gastric4) and two locations at the duodenum (See Fig 6). Surgical implantation procedures were similarly designed to past studies [8,19]. Leads were subcutaneously connected to a head connector (See Fig 6) and there were at least 10 days of recovery from surgery before the first data acquisition. All surgical and testing procedures were approved by the University of Pittsburgh Institutional Animal Care and Use Committee and were conducted following approved guidelines. All animal studies reported also followed the recommendations in the ARRIVE guidelines.

At each data acquisition session, a within-subject design included 10 minutes baseline recording (baseline) followed by 10 minutes of VNS. The VNS was a biphasic/bipolar signal, and its pulse amplitude and pulse width were set at 0.5 mA and 0.1 ms, respectively. Each animal received VNS at 10 and 30 Hz stimulation frequency on two different days resulting in a total of 14 data acquisition sessions. Utilizing the chosen VNS parameters, we did not observe any behavioral alterations. EGG was recorded down to DC with 2 KHz sample rate using Ripple Neuro’s Grapevine EMG front end (Ripple neuro, Salt Lake City, UT USA). VNS was applied using AM4100 stimulator (A-M Systems, Carlsborg, WA USA).

### Data pre-processing

**Fig 7:**
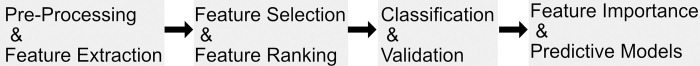
The analytic pipeline for EGG analysis and biomarker identification.

To prepare our dataset for analysis, we devised specific pre-processing steps. Our EGG recording conditions required pre-processing steps that may not be necessary when EGG is recorded sedentary. Our data were acquired from planar electrodes implanted on the serosal layer of the stomach in each ferret. Owing to the uncontrolled locomotion of the animals, which led to sporadic electrode or cable disconnections, the data acquired from each electrode exhibited varying durations and quality. Subsequent to our evaluations, we selected the signal from the electrode labeled ‘gastric2’ postulated to be proximate to the pacemaker regions of the proximal stomach [28,45] (See Fig 6). The choice of the ’gastric2’ channel was predicated on its consistent representation of the gastric slow wave signal, presumably due to its proximity to the gastric pacemaker area. While other gastric channels exhibited analogous signals, their consistency was comparatively diminished. Signals from the duodenum, which were faster, were not incorporated into this analysis, given the relatively nascent understanding of their processing compared to gastric slow wave signals. The recording methodology employed a differential input, with both the reference and recording electrodes situated similarly on the stomach’s serosal surface. Before feature engineering, we developed an in-house pre-processing pipeline, written in Python, to prepare raw signals for downstream analysis. Raw signals occasionally contained spikes with large amplitudes that are not physiologically plausible. This could have been a result of animal sudden movements or an electrode loose connection. We empirically found that a threshold level of 1e8 *μV* can remove all these spikes (See S5 and S6 Figs). A sampling frequency of 2 kHz is several orders of magnitude greater than the slow wave and spike potential responses typically observed. The EMG front end has an analog bandpass filter that can mitigate interference from unwanted frequency components. However, in scenarios where such analog filters might not be in place or when they might not be sufficiently effective, oversampling can act as an additional safeguard against aliasing. Nevertheless, given that this domain remains relatively unexplored, we elected to include frequencies up to 1 kHz in our sampling procedure [28]. This decision was made to encompass both known and potentially novel, higher-frequency signals that may be present within the data. In addition to this, by using a higher sampling rate, the quantization noise is spread over a wider frequency range [46], and then a low-pass filter can be applied to remove high-frequency noise, resulting in a cleaner signal. Finally, a higher sampling rate can help mitigate aliasing issues [47] caused by interference from other signals or noise sources. This can be particularly useful in environments, like the lab where ferrets were kept, with much electromagnetic interference or other signal disturbances. As the main frequency of EGG is in a narrow frequency band that is near to DC (0.01–0.5Hz), we decided to use a digital filtering approach called Index Blocked Discrete Cosine Transform Filtering Method [48] (IB-DCTFM). This method removes unwanted frequency range signals on the time domain by blocking specific DCT index on the DCT domain. Although like IIR filters, IB-DCTFM may cause signal distortion, such as Gibbs phenomenon [48], but in comparison to FIR and IIR counterparts, IB-DCTFM provides several advantages including superior SNR and correlation coefficient to clean signal, stability, linear phase, and zero delay. IB-DCTFM has been used as a filtering method for EGG signals [9]. The VNS artifacts in the recordings were removed using this band-pass filtering (See S7 Fig). After bandpass filtering, we applied another thresholding but this time with the threshold level set to 2000 *μV* to keep signal amplitude in a physiologically plausible range [28]. The thresholding procedure was done by substituting the values surpassing the designated threshold with the mean value of the signal amplitude. This method ensures that the signal is effectively constrained within the established bounds while maintaining its overall statistical properties. Our proposed analysis pipeline is built upon four modules (1) pre-processing and time and frequency domain feature extraction, (2) Feature selection using our proposed voting algorithm, (3) train and validate classifiers for two different classification scenarios, and (4) reporting the feature importance and classification metrics (See Fig 7).

### Feature engineering

For feature engineering, we used windows of 1-minute length with 20-seconds overlap for each 10-minute segment of our EGG signal. The choice of 1-minute length is to capture frequencies as low as 3 cycles per minute (cpm). This lower limit of 3 cpm is the reported bottom range for ferret gastric slow wave signals [28]. As the EGG signal is not stationary and has a chaotic nature, we hypothesized that dominant frequency (DF) and other features that are derived from DF, may not accurately describe the effects of VNS. Furthermore, existing research demonstrates that under conditions of dynamic and noisy EGG, particularly during rapid and unexpected movements, the selection of suitable EGG biomarkers (features) assumes heightened significance for maintaining the validity of the analysis [20]. Other fields of biosignal analysis, such as EEG or Electromyography (EMG) analysis [49,50], have developed features from both time and frequency domains that could be more suitable to extract information from non-stationary signals. In the following two sub-sections, we introduce the features that we adopted from the literature to represent 1-minute segments of the EGG signals.

### Time domain features

Time-domain features (TDFs) are derived from the amplitude of EGG signals, capturing various characteristics that reflect the underlying dynamics of the data. Previous research has demonstrated that the amplitude of EGG signals is influenced by factors such as the ingestion of food or pharmaceutical substances [17], as well as the presence of nausea [20]. Consequently, we hypothesized that the statistical distributions of EGG signals during baseline and VNS periods would exhibit differences. We calculated a group of statistical features including mean, variance, mode, median, skewness (third moment describing data asymmetry), and kurtosis (fourth moment determining tailedness of the distribution). Root mean squared value (RMS) and Line length are TDFs pertinent to signal amplitudes. Notably, Line length serves as an approximation of Katz’s fractional dimension, as described in previous literature [31]. RMS offers insights into a signal’s overall energy [21], which can facilitate differentiation between distinct signal classes or detection of particular events. For instance, research findings presented in [51] demonstrate a higher mean value or RMS during fasting as opposed to the postprandial state. The application of RMS in EGG analysis, therefore, may provide an additional perspective for understanding and interpreting data. Both RMS and Line length have been employed in EEG [31] and EGG studies [14,32], attesting to their relevance and applicability in the analysis of such signals. Fractal dimensions, including PFD [26], have been extensively employed in EEG and ECG literature [52–55], indicating that they may offer valuable insights into the complexity and self-similarity of physiological signals. We direct readers with an interest in comparing various fractal dimension methodologies to consult reference [27] for a detailed examination and comparative analysis.

Entropy is a measure of the unpredictability, complexity, or randomness of a signal or dataset [56]. Different entropy measures are related in the sense that they all quantify the complexity or randomness of a signal. Still, they do so using different approaches and algorithms. Some measures are more suitable for specific types of signals or applications. For example, approximate and sample entropy is more suitable for analyzing the regularity of time-series data, and permutation entropy is particularly useful for non-stationary signals [33]. It can be applied to study the dynamics and interactions of complex systems, such as biological systems. Entropy-based measurements serve as valuable tools for quantifying uncertainty and disorder in time series signals [33,56,57], including EGG signals [20]. Among the various entropy measures, approximate entropy and sample entropy are particularly useful for assessing the regularity and fluctuation in a time series [22]. Sample entropy has been demonstrated to be a robust feature for analyzing noisy EGG signals [20]. In addition to sample entropy, permutation entropy [33] and Singular Value Decomposition entropy (SVDEn) are employed to evaluate the local complexity and temporal- spatial complexity of a process [34], respectively. SVDEn has been employed in the examination of heart rate variability, owing to its straightforward implementation, and reduced computational complexity, particularly when analyzing short, nonstationary data series [58]. Signal variance, also known as the Hjorth activity parameter, is another time-domain feature. It indicates the surface of the power spectrum in the frequency domain [59]. Mobility and complexity, the other two Hjorth parameters [37], were also selected as time-domain features for their unique contributions. The mobility parameter is defined as the square root of the ratio of the variance of the first derivative of the signal to that of the signal itself. This parameter offers insights into the signal’s dynamic characteristics. Meanwhile, the complexity parameter reveals how similar the shape of the signal is to a pure sine wave, providing information about the signal’s waveform morphology. The value of Complexity converges to 1 as the shape of signal becomes more similar to a pure sine wave [59]. In the context of signal analysis, certain features such as RMS and entropy measures may not exhibit a direct mathematical relationship. Nevertheless, these features can be employed in conjunction to provide a comprehensive understanding of a signal’s characteristics. For instance, a high RMS value coupled with elevated entropy may be indicative of a signal characterized by significant noise and an abundance of random variations. Conversely, a high RMS value accompanied by diminished entropy could suggest the presence of a robust, periodic signal exhibiting a regular pattern.

### Frequency domain features

Analysis of frequency domain features (FDFs) is important because FDFs can provide information regarding the rhythmic patterns of signals. In the field of EGG, DF or peak frequency, is an FDF that has been widely used by researchers for EGG related analysis [28,45,60]. Dominant power (DP) or the power associated with DF is another feature often used along with DF [20]. Spectral entropy (SE) is a measure of the random process uncertainty from the frequency distribution. SE has been used to measure depth of anaesthesia using EEG [30]. A low SE value means the frequency distribution is intense in some frequency bands. Its calculation is similar to that used for the Shannon entropy, but it replaces the probability distribution with the normalized power spectral density [61] (PSD). We calculated the mean value of signal power for 3–8 cpm, 8–11 cpm, and 11–15 cpm bands equivalent to bradygastria, normogastria, and tachygastria bands [28].

There is no broad consensus on what these ranges should be in ferrets, however we chose these ranges based on methodology and findings of past studies [19,28] related to ferrets EGG. In order to calculate the PSD, we set the desired frequency resolution to 0.1 cpm and used the Welch method. Table 1 lists the time- and frequency domain features used in this paper.

### Feature selection

**Fig 8:**
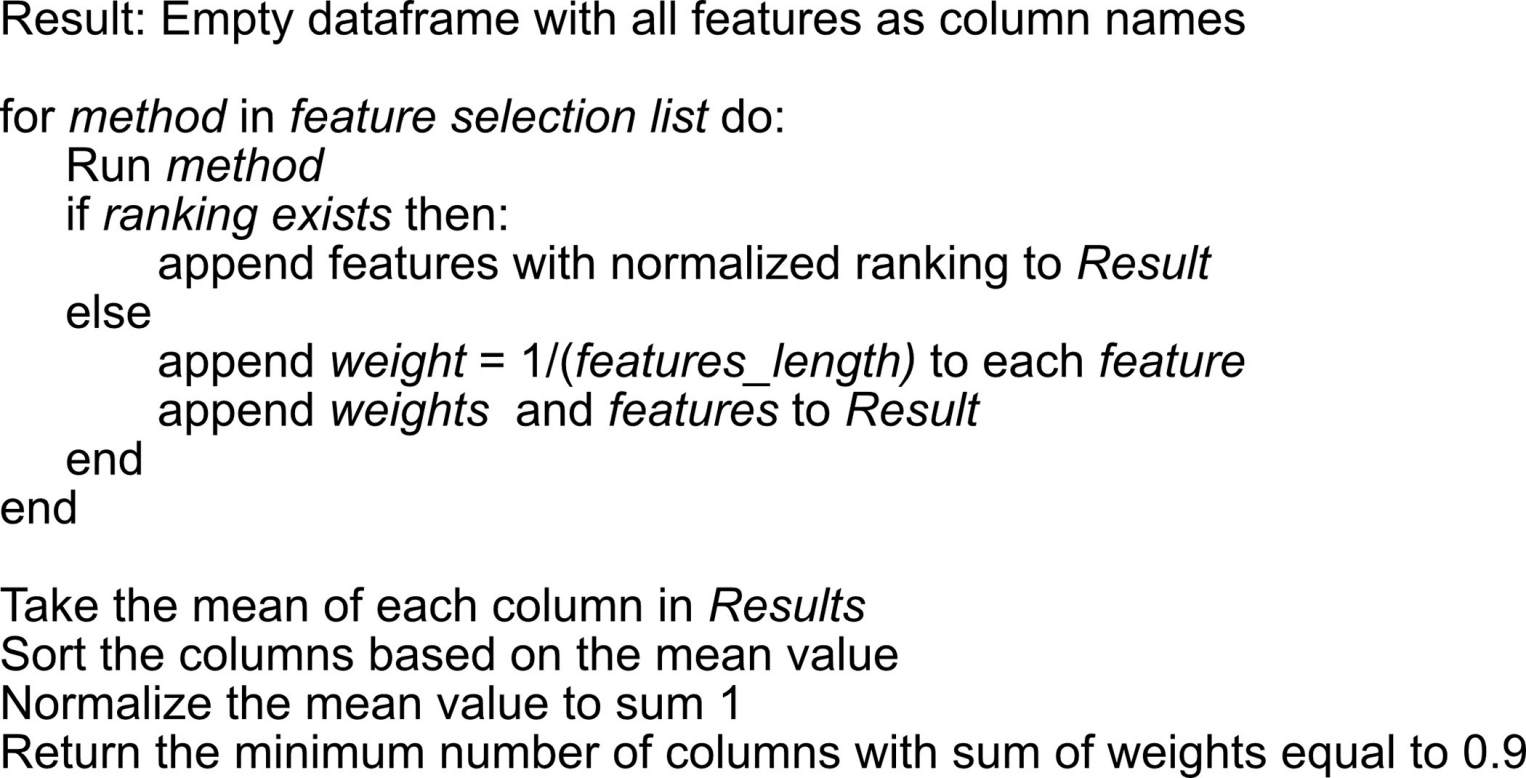
Feature selection algorithm.

As the number of features, also known as predictors (p), increases, the domain that they span increases at rates that the available data become sparse. This, in turn, requires more samples (n) to provide effective coverage of the domain for a predictive modeling problem such as classification. This concept is known as the "curse of dimensionality” [62]. As samples in high dimensional space may become equidistant, machine learning algorithms that use distance measures or other local models (in feature space) often degrade in performance as the number of features is increased [39]. In this study, we had a total of 223 samples and 29 features. For the first scenario, VNS at 10 Hz, we had 114 samples (baseline = 61, during VNS = 53). For the second scenario, VNS at 30 Hz, we had 109 samples (baseline = 51, during VNS = 58). Drawing upon features utilized in other fields, such as EEG, ECG, or EMG, the present study incorporated infrequently employed features, such as PFD 27, into EGG signal analysis. Due to the lack of prior exploration of these features in the EGG domain, it was challenging to ascertain their informativeness a priori. Consequently, the inclusion of potentially redundant or non-informative features may have an adverse effect on the performance of the classifier [40,63], necessitating further investigation and potential refinement of the feature set. To demonstrate the presence of redundancy and correlation among the features, a three-step process was undertaken. First, the Spearman correlation coefficient was computed for the features, resulting in a symmetric correlation matrix. Second, this matrix was transformed into a distance matrix by subtracting each correlation coefficient from 1. Finally, hierarchical clustering [64] was employed to group and organize the features based on their similarity. A correlation heatmap, generated using the ordered features, illustrated the extent of correlation among the engineered features by exhibiting distinct hot and cold clusters (Fig 1). As each feature selection method (Table 2) may select a different set of features with different orders [65] (importance), we proposed a voting algorithm to assign a weight to each feature. These weights are scaled to add up to one. Feature importance is a by-product of some feature selection methods such as linear regression or decision trees [23]. Additionally, we used variance thresholding that removes all low-variance features. In this case, we had no feature importance, so we assigned an equal weight to each feature, the weight being 1/ (number of selected features). Next, we calculated the average weights for all features and sorted them based on their normalized rank. Ultimately, the optimal subset of features was determined by selecting features in descending order based on their respective normalized rank, with the cumulative sum of the ranks reaching a threshold of 0.9. A threshold of 0.9 for cumulative feature importance is based on a heuristic approach to retain a majority of the information while reducing the overall dimensionality of the dataset [66] (See Fig 8).

**Table 2:** List of feature selection methods used in the voting algorithm. Except for Variance Thresholding that is independent from the target variable and hence was applied to the whole data, all the other methods were implemented using 5-fold cross validation.

**Feature selection with feature importance**
ANOVA F-value Mutual Information[39]
L1-based Linear Support Vector Classifier (LSVC) [40,65]
L2-based LSVC[40,65]
Recursive Feature Elimination (RFE) Random Forest [39,40]
Backward Sequential Feature Selection (SFS) [40,65]
Forward SFS [40,65]
Permutation Importance (PI) Random Forest [23,65]
**Feature selection without feature importance**
Variance Threshold [39]

### EGG state classification

We calculated all TDFs and FDFs listed in Table 1 for each 1-minute of the EGG signal. Following Algorithm 1, we selected the most important features for classification scenarios (See Results) and used them to train the classifiers.

### Model selection/training

The choice of classification method depends on the data and the context in which the classifier’s output will be used. Finding a classification method with the highest predictive accuracy and interpretability is challenging in practical settings, especially in datasets with small sample size. Moreover, the desired trade-off between interpretability, accuracy, and computational efficiency also plays a crucial role in determining the appropriate method for a given task [67].

There are advantages and disadvantages to each classification model under different circumstances. Decision trees are relatively fast and useful if one needs to share the results with an audience interested in how a conclusion was reached, however, they tend to overfit [68]. Support vector machines (SVM) are another choice for binary classification. They often provide high accuracy and tend not to overfit the data. Linear SVMs, as opposed to their non-linear counterparts, produce a linear decision boundary that can be easily understood and visualized [69]. However, the practitioners need to spend time training and tuning SVMs up front. Artificial neural networks (ANNs) are powerful for modeling nonlinear data with a high number of input features. However, ANNs can become computationally expensive. As the number of nodes and layers increase, it is difficult to interpret how an ANN has reached its solution and fine-tuning an ANN may involve multiple steps and hyperparameters. In our case, with a rather small number of observations and large number of features, it is crucial to select models that can effectively handle high-dimensional data and mitigate the risk of overfitting. To identify the best model, we took an empirical approach to test and discover which classifier achieves the best classification performance [70]. Considering the limitations of our dataset (small sample size, large number of features) and the research questions we aimed to answer, we used Random Forest classifiers [23], SVM [69] (with linear and radial kernel), Naïve Bayes classifier [71], and logistic regression classifier for our binary classification tasks. Our preliminary experiments showed that Random Forest was consistently performing better than the other classifiers we used. (See S8 and S9 Figs)

The model selection procedure was as follows: Data were divided into training and test sets in a stratified manner to keep the ratio of baseline (class0) to VNS (class1) the same for both training and test data. 80% of the whole data was used for training, and the rest was used at the inference step. All models were trained with their default parameters and evaluated using stratified 5-fold cross validation (CV). The shuffle parameter of cross-validation was set to False, to maintain the original sequence of the samples. After the initial training, we selected the best classifier based on its performance and tuned its hyper parameters. Tuning was implemented by utilizing a hyper parameter optimization framework named Optuna [72]. Optuna allows for dynamic construction of the search space and provides a combination of an efficient searching and pruning algorithm to speed up optimization.

### Model evaluation

In this study, we utilized several evaluation metrics, including accuracy, ROC-AUC, f1-score, and f2-score, to assess the performance of our models. In our dataset, there exists an imbalance in the distribution of samples. Specifically, in the context of the VNS at 10 Hz scenario, we observe an approximate surplus of 15% in VNS samples relative to the baseline. Similarly, in the VNS at 30 Hz scenario, the excess of VNS samples over the baseline is approximately 14%. The ROC-AUC has been demonstrated to be a measure of choice for assessing the performance of a classifier for imbalanced data [24,73]. Consequently, we have chosen to highlight AUC as the primary metric in our results, while providing additional metrics such as accuracy, f1-score, and f2-score in the supplementary materials for further reference. To prove that our trained classifier has a ROC-AUC score significantly higher than chance level (0.5), we conducted a permutation test [74,75]. We first trained the classifier on the original dataset and computed its ROC-AUC scores using 5-fold CV. Following this, we performed a permutation test by randomly shuffling the true labels of the dataset, retrained the classifier on this permuted dataset, and obtained the ROC-AUC scores of 5-fold CV for each shuffle. This procedure was repeated 1000 times to generate a distribution of permuted ROC-AUC scores. Next, we compared the ROC-AUC scores of the trained classifier on the original dataset to the distribution of permuted ROC-AUC scores by calculating the p-value, using a two-sample Kolmogorov-Smirnov (KS) test [25]. KS test is a non-parametric test that is sensitive to variations in both the location and shape of the empirical cumulative distribution functions pertaining to the two samples under consideration. If the p-value was found to be below 0.05 significance level, we could reject the null hypothesis and conclude that the trained classifier exhibited a ROC-AUC score significantly higher than 0.5, indicating its performance surpasses random guessing.

## Appendix

The Petrosian Fractal Dimension (PFD) [26,27] is a method designed to measure the complexity or irregularity of a signal and is computationally efficient compared to other traditional fractal dimension estimation techniques. The subsequent equation illustrates the computation of the Petrosian Fractal Dimension:

$$PFD=\frac{\log_{10}N}{\log_{10}N+\log_{10}\left(\frac{N}{N+0.4N\delta }\right)}$$

where N is the window length, and N*δ* is the number of sign changes in the signal derivative.

## Supporting information

S1 Fig Comparison of the RMS values. a) baseline, b) VNS at 10 Hz, c) VNS at 30 Hz.
(DOCX)

S2 Fig Performance of the trained Random Forest for the first scenario. a) Accuracy, b) F1-score, c) F2-score.
(DOCX)

S3 Fig Comparison of the DF values. a) baseline, b) VNS at 10 Hz, c) VNS at 30 Hz.
(DOCX)

S4 Fig Performance of the trained Random Forest for the second scenario. a) Accuracy, b) F1-score, c) F2-score.
(DOCX)

S5 Fig Effect of pre-processing steps. a) Raw baseline signal, b) Baseline signal after thresholding, c) Baseline signal after band-pass (bandpass cutoffs: 0.01–0.5Hz) filtering.
(DOCX)

S6 Fig Effect of pre-processing steps. a) Raw VNS at 10 Hz signal, b) VNS signal after thresholding, c) VNS signal after band-pass (bandpass cutoffs: 0.01–0.5Hz) filtering.
(DOCX)

S7 Fig Impact of the pre-processing pipeline on the elimination of artifacts induced by VNS. Panels a) and b) illustrate the raw and pre-processed baseline data, respectively, while panels c) and d) present the raw and pre-processed VNS data, correspondingly.
(DOCX)

S8 Fig Classification performance of the Random Forest trained with the selected features of VNS at 10 Hz against SVM and Gaussian Naïve Bayes (GNB). (DOCX)

S9 Fig Classification performance of the Random Forest trained with the selected features of VNS at 30 Hz against SVM and Gaussian Naïve Bayes (GNB). (DOCX)
