# Supplementary material for: Quantifying the effects of vagus nerve stimulation on gastric myoelectric activity in ferrets using an interpretable machine learning approach
Source: PLoS One. 2023 Dec 1;18(12):e0295297. doi: 10.1371/journal.pone.0295297 (PMC10691721; doi:10.1371/journal.pone.0295297)
Supplement: S1 Fig — a) baseline, b) VNS at 10 Hz, c) VNS at 30 Hz. (DOCX) [file pone.0295297.s001.docx]

Figure S1 demonstrates the histogram plot of bootstrapped RMS values of baseline, VNS at 10 Hz, and VNS at 30 Hz. RMS values of baseline were statistically significantly different from VNS at 10 Hz and VNS 30 Hz (p-values < 0.001 and t-statistic = 118.862 and -69.637, respectively). The difference between RMS values of baseline and VNS at 10 Hz was greater than of baseline and VNS at 30 Hz. (52.82 µV vs -33.42 µV)


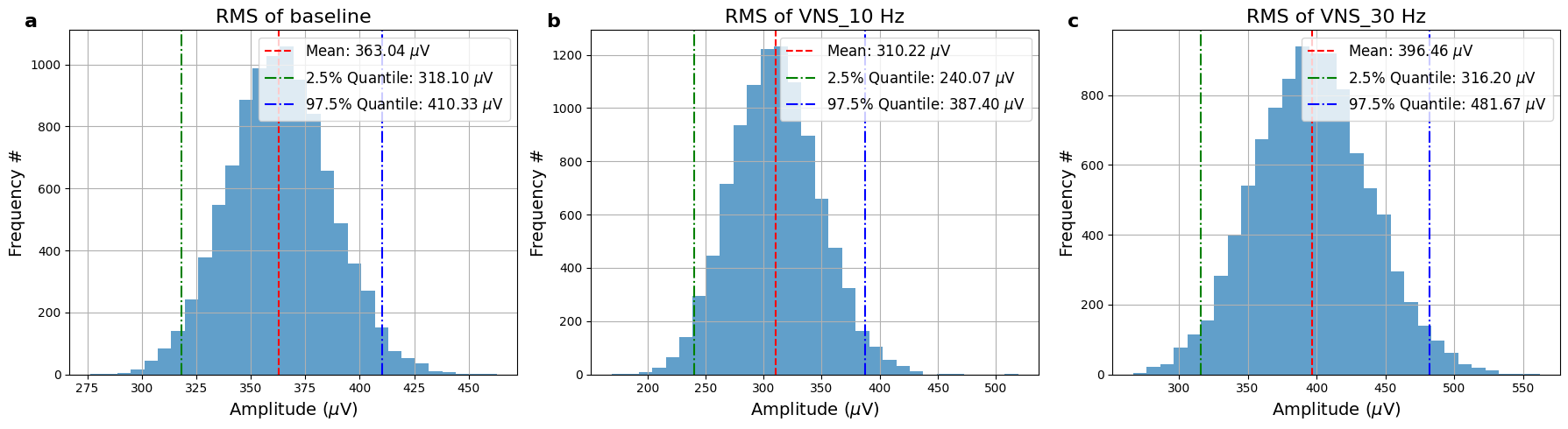


Figure S 1 Comparison of the RMS values. a) baseline, b) VNS at 10 Hz, c) VNS at 30 Hz.
