# Supplementary material for: Quantifying the effects of vagus nerve stimulation on gastric myoelectric activity in ferrets using an interpretable machine learning approach
Source: PLoS One. 2023 Dec 1;18(12):e0295297. doi: 10.1371/journal.pone.0295297 (PMC10691721; doi:10.1371/journal.pone.0295297)
Supplement: S3 Fig — a) baseline, b) VNS at 10 Hz, c) VNS at 30 Hz. (DOCX) [file pone.0295297.s003.docx]

Figure S3 demonstrates the histogram plot of bootstrapped dominant frequency (DF) values of baseline, VNS at 10 Hz, and VNS at 30 Hz. DF values of baseline were statistically significantly different from VNS at 10 Hz and VNS 30 Hz (p-values < 0.001 and t-statistic = -106.132 and -198.124, respectively). The difference between DF values of baseline and VNS at 30 Hz was greater than of baseline and VNS at 10 Hz. (-1.05 cpm vs -0.59 cpm)


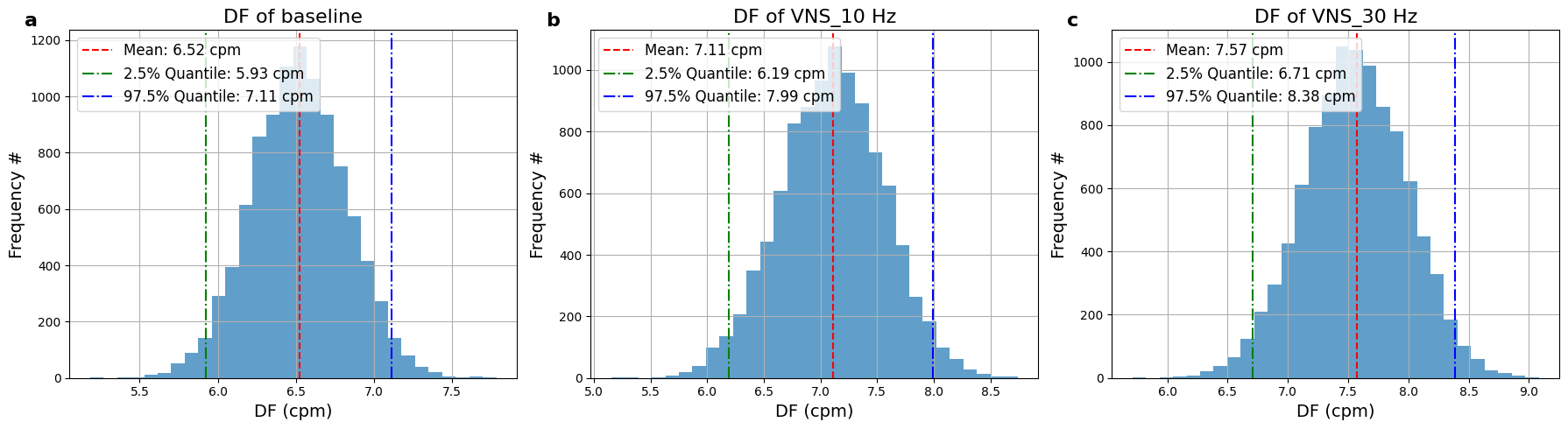


Figure S 3 Comparison of the DF values. a) baseline, b) VNS at 10 Hz, c) VNS at 30 Hz.
