# Supplementary material for: Quantifying the effects of vagus nerve stimulation on gastric myoelectric activity in ferrets using an interpretable machine learning approach
Source: PLoS One. 2023 Dec 1;18(12):e0295297. doi: 10.1371/journal.pone.0295297 (PMC10691721; doi:10.1371/journal.pone.0295297)
Supplement: S4 Fig — a) Accuracy, b) F1-score, c) F2-score. (DOCX) [file pone.0295297.s004.docx]

Figure S4 demonstrates the accuracy, f1-, and f2-score of the trained Random Forest for the second scenario (baseline vs. VNS at 30 Hz).


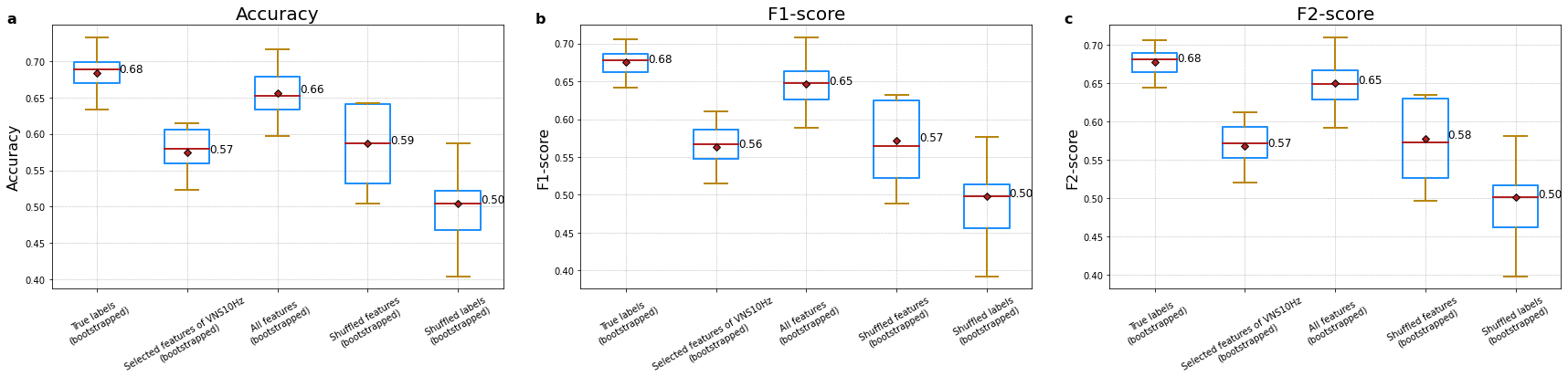


Figure S 4 Performance of the trained Random Forest for the second scenario. a) Accuracy, b) F1-score, c) F2-score.
