# Supplementary material for: Quantifying the effects of vagus nerve stimulation on gastric myoelectric activity in ferrets using an interpretable machine learning approach
Source: PLoS One. 2023 Dec 1;18(12):e0295297. doi: 10.1371/journal.pone.0295297 (PMC10691721; doi:10.1371/journal.pone.0295297)
Supplement: S5 Fig — a) Raw baseline signal, b) Baseline signal after thresholding, c) Baseline signal after band-pass (bandpass cutoffs: 0.01–0.5Hz) filtering. (DOCX) [file pone.0295297.s005.docx]

Figures S5 and S6 demonstrate the effect of pre-processing steps on a sample signal from baseline and VNS at 10 Hz, respectively.


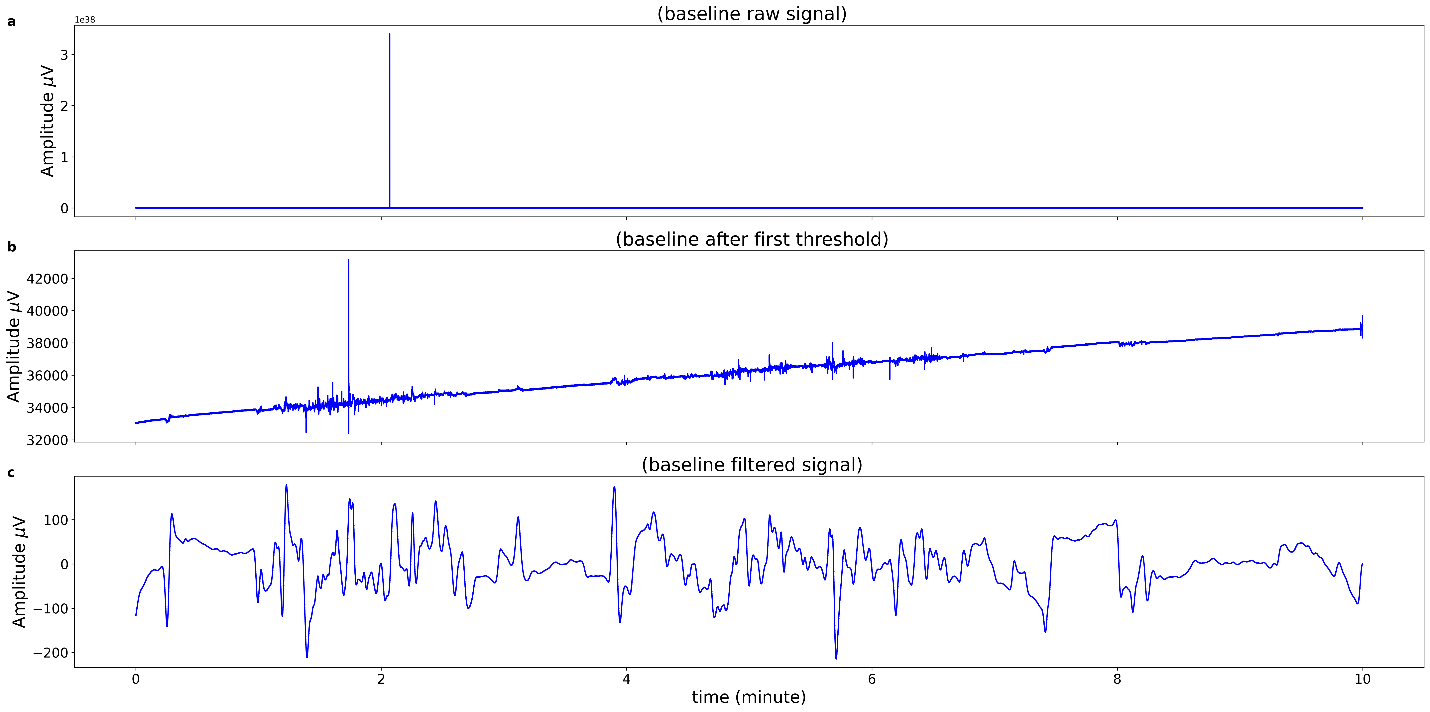


Figure S 5 Effect of pre-processing steps. a) Raw baseline signal, b) Baseline signal after thresholding, c) Baseline signal after band-pass (bandpass cutoffs: 0.01-0.5Hz) filtering.
