# Supplementary material for: Quantifying the effects of vagus nerve stimulation on gastric myoelectric activity in ferrets using an interpretable machine learning approach
Source: PLoS One. 2023 Dec 1;18(12):e0295297. doi: 10.1371/journal.pone.0295297 (PMC10691721; doi:10.1371/journal.pone.0295297)
Supplement: S6 Fig — a) Raw VNS at 10 Hz signal, b) VNS signal after thresholding, c) VNS signal after band-pass (bandpass cutoffs: 0.01–0.5Hz) filtering. (DOCX) [file pone.0295297.s006.docx]

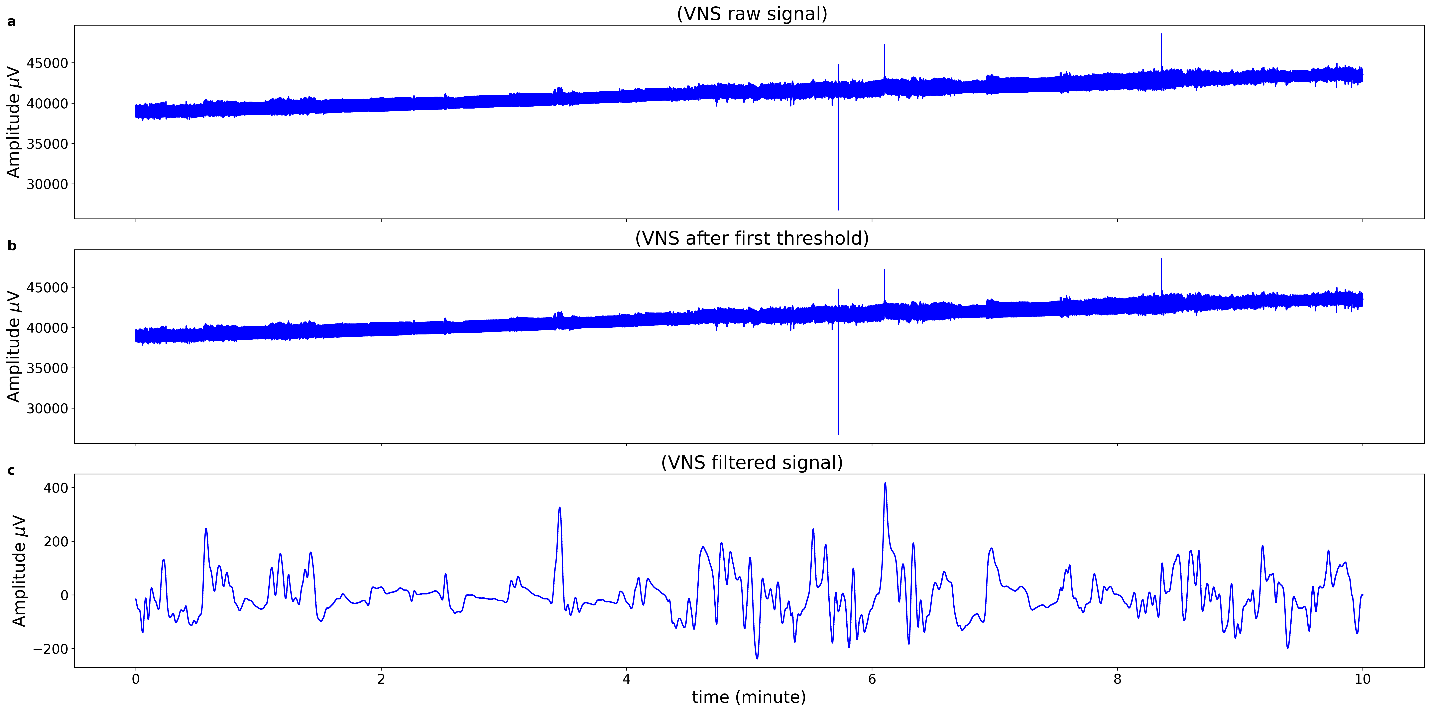


Figure S 6 Effect of pre-processing steps. a) Raw VNS at 10 Hz signal, b) VNS signal after thresholding, c) VNS signal after band-pass (bandpass cutoffs: 0.01-0.5Hz) filtering.
