# Supplementary material for: Quantifying the effects of vagus nerve stimulation on gastric myoelectric activity in ferrets using an interpretable machine learning approach
Source: PLoS One. 2023 Dec 1;18(12):e0295297. doi: 10.1371/journal.pone.0295297 (PMC10691721; doi:10.1371/journal.pone.0295297)
Supplement: S7 Fig — Panels a) and b) illustrate the raw and pre-processed baseline data, respectively, while panels c) and d) present the raw and pre-processed VNS data, correspondingly. (DOCX) [file pone.0295297.s007.docx]

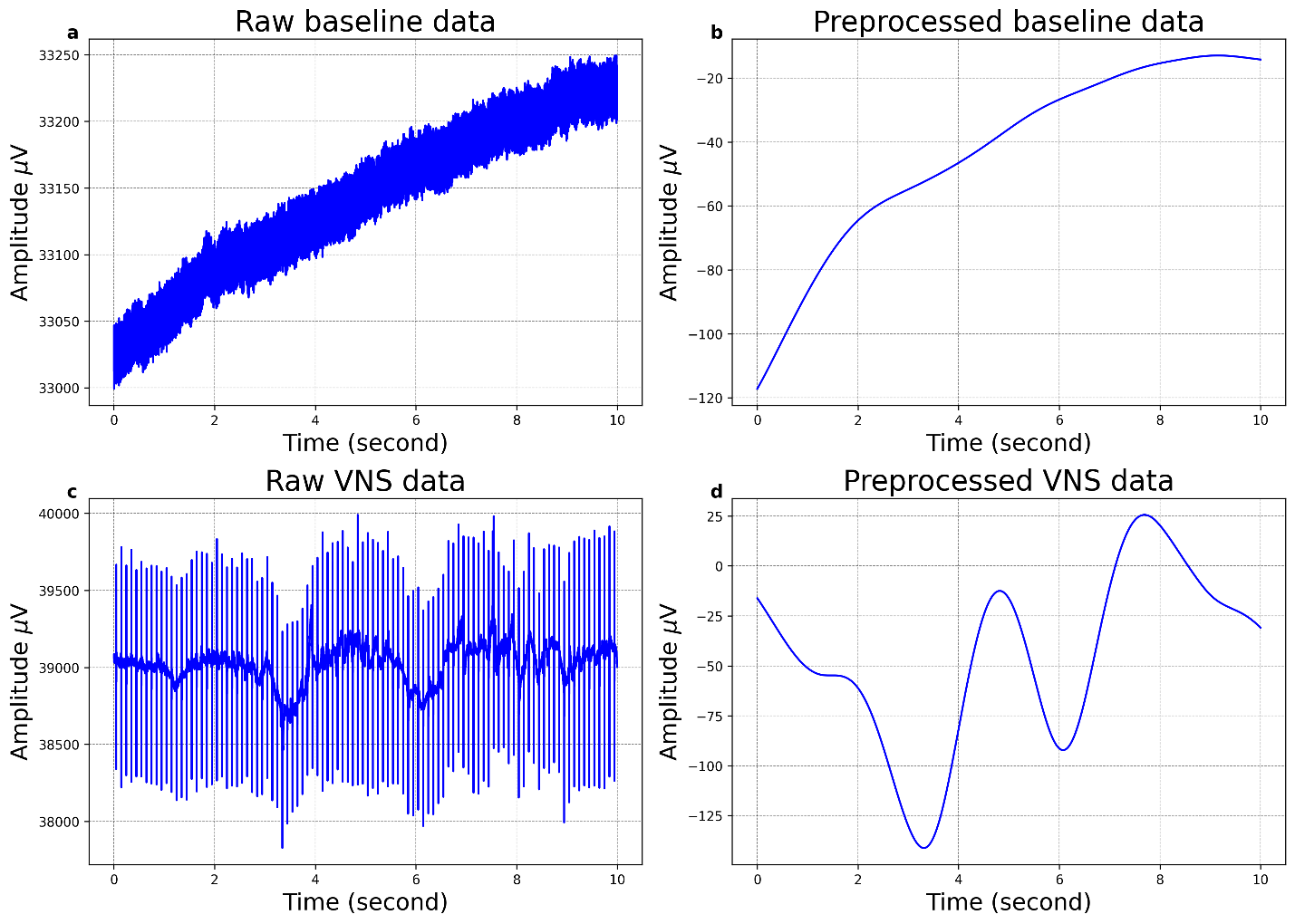


Figure S 7 Impact of the pre-processing pipeline on the elimination of artifacts induced by VNS. Panels a) and b) illustrate the raw and pre-processed baseline data, respectively, while panels c) and d) present the raw and pre-processed VNS data, correspondingly.
