# Supplementary material for: Quantifying the effects of vagus nerve stimulation on gastric myoelectric activity in ferrets using an interpretable machine learning approach
Source: PLoS One. 2023 Dec 1;18(12):e0295297. doi: 10.1371/journal.pone.0295297 (PMC10691721; doi:10.1371/journal.pone.0295297)
Supplement: S8 Fig — (DOCX) [file pone.0295297.s008.docx]

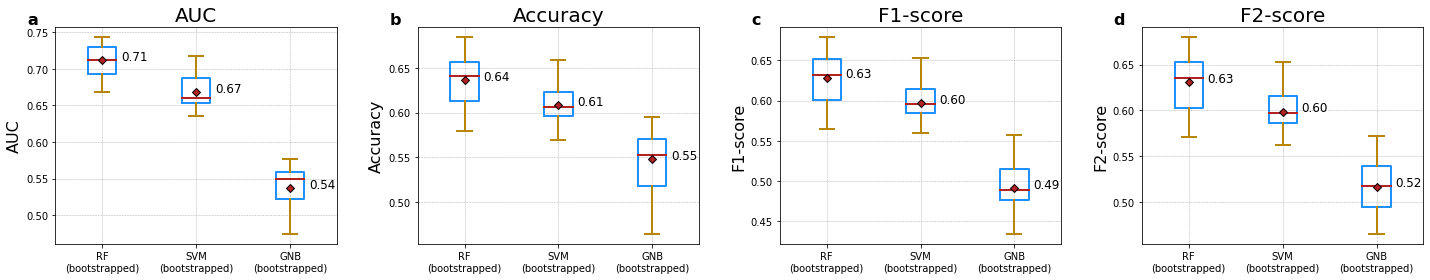


Figure S 8 Classification performance of the Random Forest trained with the selected features of VNS at 10 Hz against SVM and Gaussian Naïve Bayes (GNB).
